# Music improves the therapeutic effects of bevacizumab in rats with glioblastoma: Modulation of drug distribution to the brain

**DOI:** 10.3389/fonc.2022.1010188

**Published:** 2022-10-13

**Authors:** Oxana Semyachkina-Glushkovskaya, Sergey Diduk, Eroshova Anna, Dosadina Elina, Kruglov Artem, Alexander Khorovodov, Alexander Shirokov, Ivan Fedosov, Alexander Dubrovsky, Inna Blokhina, Andrey Terskov, Nikita Navolokin, Arina Evsukova, Daria Elovenko, Viktoria Adushkina, Jürgen Kurths

**Affiliations:** ^1^ Humboldt University, Institute of Physics, Berlin, Germany; ^2^ Deparment of Biology, Saratov State University, Saratov, Russia; ^3^ Laboratory of Pharmaceutical Biotechnology, Pushchino State Institute of Natural Science, Pushchino, Russia; ^4^ Department of Biotechnology, Leeners LLС, Moscow, Russia; ^5^ Institute of Biochemistry and Physiology of Plants and Microorganisms, Saratov Scientific Centre of the Russian Academy of Sciences (IBPPM RAS), Saratov, Russia; ^6^ Department of Pathological Anatomy, Saratov Medical State University, Saratov, Russia; ^7^ Potsdam Institute for Climate Impact Research, Department of Complexity Science, Potsdam, Germany

**Keywords:** blood-brain barrier, brain drug delivery, bevacizumab, rat glioblastoma, music

## Abstract

**Background:**

The development of new methods for modulation of drug distribution across to the brain is a crucial step in the effective therapies for glioblastoma (GBM). In our previous work, we discovered the phenomenon of music-induced opening of the blood-brain barrier (OBBB) in healthy rodents. In this pilot study on rats, we clearly demonstrate that music-induced BBB opening improves the therapeutic effects of bevacizumab (BZM) in rats with GBM *via* increasing BZM distribution to the brain along the cerebral vessels.

**Methods:**

The experiments were performed on Wistar male rats (200–250 g, n=161) using transfected C6-TagRFP cell line and the loud rock music for OBBB. The OBBB was assessed by spectrofluorometric assay of Evans Blue (EB) extravasation and confocal imaging of fluorescent BZM (fBZM) delivery into the brain. Additionally, distribution of fBZM and Omniscan in the brain was studied using fluorescent and magnetic resonance imaging (MRI), respectively. To analyze the therapeutic effects of BZM on the GBM growth in rats without and with OBBB, the GBM volume (MRI scans), as well as immunohistochemistry assay of proliferation (Ki67 marker) and apoptosis (Bax marker) in the GBM cells were studied. The Mann–Whitney–Wilcoxon test was used for all analysis, the significance level was p < 0.05, n=7 in each group.

**Results:**

Our finding clearly demonstrates that music-induced OBBB increases the delivery of EB into the brain tissues and the extravasation of BZM into the brain around the cerebral vessels of rats with GBM. Music significantly increases distribution of tracers (fBZM and Omniscan) in the rat brain through the pathways of brain drainage system (perivascular and lymphatic), which are an important route of drug delivery into the brain. The music-induced OBBB improves the suppressive effects of BZM on the GBM volume and the cellular mechanisms of tumor progression that was accompanied by higher survival among rats in the GBM+BZM+Music group *vs*. other groups.

**Conclusion:**

We hypothesized that music improves the therapeutic effects of BZM *via* OBBB in the normal cerebral vessels and lymphatic drainage of the brain tissues. This contributes better distribution of BZM in the brain fluids and among the normal cerebral vessels, which are used by GBM for invasion and co-opt existing vessels as a satellite tumor form. These results open the new perspectives for an improvement of therapeutic effects of BZM *via* the music-induced OBBB for BZM in the normal cerebral vessels, which are used by GBM for migration and progression.

## Introduction

The blood-brain barrier (BBB) due to semipermeable property limits the delivery of vast majority of cancer therapeutics, including monoclonal antibodies, antibody-drug conjugates, and hydrophilic molecules (1). However, the role of BBB in limiting drug delivery into the brain in patients with glioblastoma (GBM) is controversial (2). There is a generally accepted view that GBM is characterized by disruption of BBB for the high- and low-molecular-weight substances (3–5). Nevertheless, there is oppositely overwhelming clinical evidence suggesting migration of GBM *via* an intact BBB colonizing the normal cerebral vessels (2, 3). Most clinical data clearly demonstrate that GBM patients have an intact BBB around tumor, and GBM cure will only be possible if these tumor regions are extensively treated (2, 6). Indeed, surgical data demonstrate that GBM borders exist beyond tumor volume defined radiographically that supports the concept of GBM as a whole-brain disease (7). Therefore, an important first step in the effective GBM therapy is the development safe methods bypassing an intact BBB at the border of GBM to prevent tumor migration and progression (2).

Over the past 30 years, Food and Drug Administration (FDA) proposed only four medications for the GBM therapy, such as lomustine, carmustine, temozolomide (TMZ), which are alkylating agents, and bevacizumab (BZM), which is a monoclonal antibody preventing activation the vascular endothelial growth factor (VEGF) and dfi 2emonstrating antiangiogenic effects (8–10). The GBM development, depending on its angiogenesis has been provided, has a high potential for antiangiogenic cancer therapy (11, 12). Antiangiogenic therapy works through vascular normalization and improvement of delivery of chemotherapy (13–19). However, there is clinical evidence indicating that BZM controls edema effectively in GBM patients, but BMZ does not demonstrate a convincing impact on their survival (15, 16, 20, 21). The mechanisms of BZM failure are complex, including the inability to deliver BZM to the normal cerebral vessels through which GBT migrates (21). Indeed, animal data provide that antiangiogenic therapy using the VEGF blockade does not prevent GBM invasion of normal cerebral vessels leading to GBM spreading and progression (22). Therefore, the modulation of BZM delivery into the normal cerebral vessels can significantly improve the therapeutic effects of antiangiogenic therapy of GBM.

In our previous study, we developed a non-invasive method of sort-lasting and reversible BBB opening in the normal cerebral vessels of rodents induced by loud rock music (23) or sound (24). We clearly demonstrate that music-induced BBB opening can have a high potential for clinical applications, especially for GBM therapy because the BBB opens in unspecified manner in 11 regions of the brain that might be important for the treatment of GBM, which is characterized by diffusive progression without specificity of the brain areas. Furthermore, music is easily used, non-invasive, low cost, labeling free, perspective, and completely new approach for GBM therapy.

In this pilot study, we investigate the music-induced distribution of BZM along the cerebral vessels in rats with GBM and analyzed the progression of GBM in the treated and untreated groups with anti-VEGF antibody and the music-induced BBB opening.

## Material and methods

### Subjects and groups

Pathogen-free male Wistar rats (200–250g, 2 months old) were used in all experiments and were obtained from the National Laboratory Animal Resource Centre (Pushchino, Russia). The animals were housed under standard laboratory conditions with access to food and water ad libitum. All experimental procedures were performed in accordance with the “Guide for the Care and Use of Laboratory Animals”, Directive 2010/63/EU on the Protection of Animals Used for Scientific Purposes, and the guidelines from the Ministry of Science and High Education of the Russian Federation (Nº 742 from 13.11.1984), which have been approved by the Bioethics Commission of the Saratov State University (Protocol No. 7). The experiments were performed in the following groups: (1) GBM, the group included rats with GBM and received intraperitoneal injection of physiological saline solution (PSS); (2) GBM+Music, the group included rats with GBM and the music-induced BBB opening; (3) GBM+BZM, the group included rats with GBM and received intraperitoneal injection of BZM; (4) GBM+BZM+Music, the group included rats with GBM treated by music and received intraperitoneal injection of BZM; (5) Healthy group (HG), the group included healthy sham rats with the injection of PSS in the same volume and in the same area of the brain, which was selected for the injection of glioma cells; (6) HG + Music, the group included the healthy intact rats with the music-induced BBB opening; n=7 in each group.

**Figure 1:**
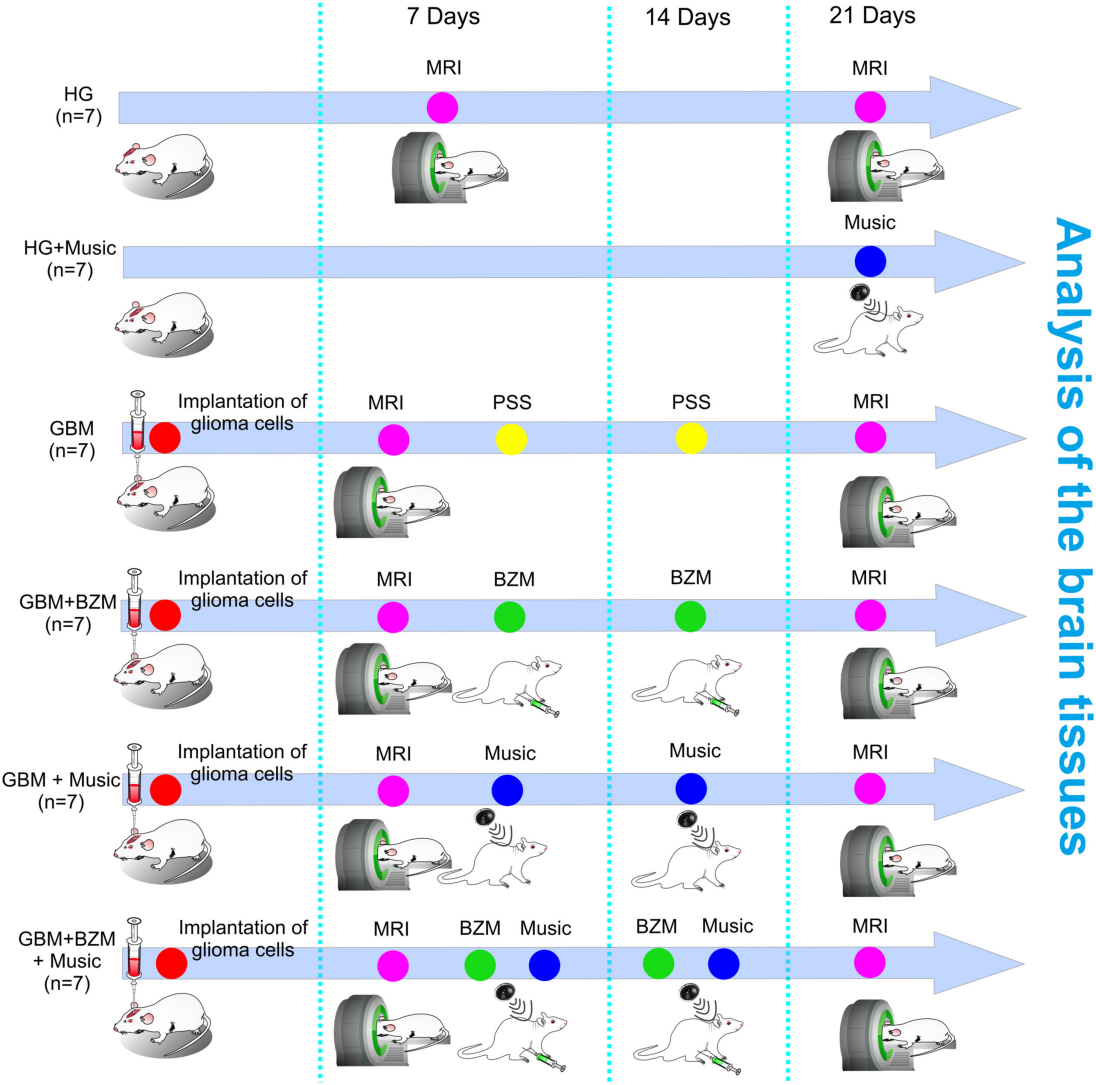
Schematic illustration of the design of the experiments. The rats were divided into six groups: (1) GBM, the group included rats with GBM and received intraperitoneal injection of PSS; (2) GBM+Music, the group included rats with GBM and the music-induced BBB opening; (3) GBM+BZM, the group included rats with GBM and received intraperitoneal injection of BZM; (4) GBM+BZM+Music, the group included rats with GBM treated by music and received intraperitoneal injection of BZM; (5) HG, the group included healthy sham rats with the injection of PSS in the same volume and in the same area of the brain, which was selected for the injection of glioma cells; (6) HG + Music, the group included the healthy rats with the music-induced BBB opening; n=7 in each group. The PSS and BZM (10 mg/kg) intraperitoneal injection were performed 7 days and 14 days after the implantation of glioma cells into the rat brain. The MRI analysis of GBM growth and the tumor volume was performed 7 and 21 days post tumor cell implantation. The rats from the GBM+Music and the GBM+BZM+Music groups listened music on 7 and 14 days of tumor growth; rats from the HG group listened music on 21 day of observation. GBM, glioblastoma; BZM, bevacizumab; HG, healthy group; MRI, magnetic resonance imaging; PSS, physiological saline solution.

**Figure 1** represents the design of the experiments. Rats with GBM were treated with BZM and music alone as well as with their combination. The BZM was injected in dose 10 mg/kg following the FDA recommendations (8–10). The first dose of BZM was given 7 days after the implantation of glioma cells when the initial MRI sign of brain tumor was detected. The second dose of BZM rats received 14 days of glioma growth in accordance with FDA guidance (8).

### Implantation of C6-TagRFP glioma

The rats were pre-treated by pre-medication with Seduxen (Gedeon Richter, Hungary) in a dose of 50 µg/ml. Afterward, rats were deeply anesthetized with intraperitoneal Zoletil (Virbac, France) in a dose of 100 µg/kg and moved into a stereotaxic head holder and immobilized on the stereotaxic system (Stoelting, New York, US) by fixation of the head. The scalp of the anesthetized rat was shaved and scrubbed with betadine three times followed by an alcohol rinse. Hair was removed at the site of the planned operation and a cut was made in the area of the planned injection. An incision was made over the sagittal crest from the bregma to the lambdoidal suture and periosteal membrane was removed. A small dental drill was used to create a burr hole through the bone without tearing the dura matter in the exposed cranium 0.5 mm anterior and 3 mm lateral to the bregma.

The cell line of rat glioma (C6) was obtained from the Russian Cell Culture Collection of Vertebrates, Institute of Cytology, Russian Academy of Sciences, (St. Petersburg, Russia). A transfected C6-TagRFP cell line was used for the study of the growth of fluorescent GBM (25). C6 cells were cultured in a Dulbecco′s Modified Eagle′s Medium (Paneco, Russia) containing 2.5% of fetal bovine serum (FBS, Biosera), 4 mM glutamine (Paneco, Russia), penicillin (50 IU/ml), and streptomycin (50 mg/ml) (Paneco, Russia). C6 cells were transfected with TagRFP-CDNA plasmid using the method of liposomal transfection followed by selection using geneticin (G418 antibiotic, neomycin analogue). The resulting cell line C6-TagRFP has stable cultural and morphological characteristics.

The glioma cells (5×10^5^ cells per rat) were injected at a depth of 4.5 mm from the brain surface into the caudate putamen area with a Hamilton microsyringe (25 μl, Hamilton Co, Reno, Nevada, US) with the needle (25 Gauge) in a volume of 15 µl at a rate of 3 µl/min using the stereotaxic manipulator (Stoelting, New York, US). The duration of implantation did not exceed 10–15 min. The PSS (15 µl, Sigma-Aldrich, US) was injected in the same region of the brain in the sham groups. Thereafter, the burr hole was sealed with sterile bone wax and tissue glue and the wound sutured closed with 3-0 absorbable suture material. After the implantation of glioma cells, the wound was closed and treated with 2% brilliant green dye solution. The rats were removed from the stereotaxic head holder, given 0.01 mg/kg buprenorphine, s.c., and 50,000 units bicillin, i.m., and returned to their cages after recovery in the temperature-controlled recovery cage and moved back to animal facility after recovery. The animal was placed in a clean cage. The growth of fluorescent GBM was assessed also by confocal microscopy using the Leica SP5 confocal laser scanning microscope (Leica, Germany) and 3D images were obtained using the LAS x software platform of the LSX life Science microscope.

### Synthesis of fluorescent BZM

The BZM was labeled with the Fluorescein isothiocyanate (FITC) (Invitrogen, Waltham, Massachusetts, US). The BZM (11 g/l) dissolved in the storage buffer (9 mM Na2HPO4, 42mM NaH2PO4*H20, pH 6.18) and diluted with sodium carbonate buffer pH 9.0. The FITC (20 mg/ml) was dissolved in the dimethyl sulfoxide (Sigma-Aldrich, Saint Louis, Missouri, US) and added to BZM solution in mass ratio 74:1. The 3 h after incubation at room temperature in dark place with occasional stirring by pipetting, unbounded BZM was removed by buffer exchange using Amicon Ultra centrifuge filter (30 kDa). The labeled BZM was sterile filtered through 0.20 µm pore size PES membrane filter, diluted with storage buffer to final concentration of 10 g/l and stored in light-protected plastic tube at 2–8°C. The molecular weight of FITC-BZM was 149 kDa.

### Magnetic resonance imaging of GBM

A tumor volume assessment was performed 7 and 21 days post–tumor cell implantation into the rat brain using a seven Tesla dedicated research MRI scanner (Bruker BioSpin; Billerica, MA, USA). To obtain a good signal-to-noise ratio, a 72 mm small-bore linear RF volume coil with an actively decoupled brain surface coil (40 cm bore; a 660 mT/m, rise time within 120 μs) was used for excitation and signal detection, respectively. Rats were anaesthetized with 2% isoflurane at 1 L/min N_2_O/O_2_—70:30 (Isoflurane, Baxter Healthcare Corp., USA) using an anesthesia machine (the Univentor 400 Anesthesia Unit, Univentor, Malta). The animal was placed into a stereotaxic headset and onto an animal holder with a surface coil. Temperature and respiration were to be monitored and maintained by thermal air blower. Anatomical T2-weighted images were acquired with a fast spin-echo sequence [rapid acquisition with relaxation enhancement (RARE)] [Repetition Time (TR)/Echo Time (TE) = 5.000 ms/56 ms, Field of View (FOV) = 4 cm×4 cm, slice thickness = 1 mm, slice gap (inter-slice distance) = 1.1 mm, number of slices = 12, matrix = 256×256, number of averaging = 3] as previously described (26). T1-weighted imaging used the RARE technique with 9.6 ms TE, 1000 ms TR, a RARE factor of 2, thus 4 averages requiring 4 min 16 s to assess tumor volumes, region of interests (ROIs) were drawn around regions of visible hyperenhancement in each of the slices on T2-weighted and corresponding T1-weighted images, using NIH ImageJ and calculated using MATLAB software (MathWorks, Inc., Natick, MA, USA) (26).

### The music-induced BBB opening

To produce music (100 dB and 11–10.000 Hz, Scorpions “Still loving you”) we used loudspeaker (ranging of sound intensity—0-130 dB, frequencies—63-15.000 Hz; 100 V, Yerasov Music Corporation, Saint Petersburg, Russia). The repetitive music exposure was performed using the sequence of: 1 min—music on, then 1 min—music off during 2 h. The sound level was measured directly in a cage of animals using the sound level meter (Megeon 92130, Russia). In our previous study, we have shown that the intensity of sound (100 dB) and its duration (2 h) but not sound frequencies (11–10,000 Hz) affect the BBB permeability (23, 24). Therefore, we did not pay attention on consistent beat and varying intensity and softer parts of rock ballad. The entire 60 min were played consecutively without special selection of one music part (i.e., 60 times 1 min until completion of the entire song). We did not analyze influences of different types of sound (pink and white noise, etc.) on the BBB permeability and focused only on music effects on the BBB permeability since music is used as therapeutic method in cognitive components and can be easily performed approach in clinical practice (27).

This protocol is based on our previous study of phenomenon of music-induced BBB opening in mice (23) and rats (24). In these studies, we demonstrated that the listening of loud rock music (90–100 dB, 11–10.000 Hz, Scorpions “Still loving you”) is accompanied by delayed (1 h after music exposure) and short-lasting (during 1–4 h) BBB opening in the normal cerebral vessels. Music-induced BBB opening for high molecular weight molecules, such as Evans Blue Albumin complex (EBAC) 68.5 kDa and FITC labeled dextran 70 kDa, has been clearly shown in *ex vivo* and *in vivo* experiments using conventional fluorescence microscopy and two-photon laser scanning microscopy. Moreover, using MRI, we have found music-induced BBB opening for low-molecular-weight molecules, such as gadolinium-diethylene-triamine-pentaacetic acid (928 Da). Note, that mice and rats have hearing with a frequency sensitivity 1–90 kHz (for mice) (28) and 8–38 kHz (for rats) (29), i.e., music used in our experiments was audible for them.

We have chosen the intermittent music treatment (1 min—sound; 1 min—pause) because the safe listening time for a continuous sound of 100 dB is 15 min (30). However, it has been no effect found on the BBB permeability when animals listened continues music (100 dB) during 15 min, i.e., without intermittent algorithm (1 min—sound, 1 min—pause) [see, SI in (24)]. Therefore, the longer music impact (1 h and 2 h) in a repetitive mode has been chosen for an adaptation to loud sound. When stimuli are repeated, neural activity is adapted (31) on the level of individual cortical neurons and hemodynamic changes (32–34). A stimulus period of 2 h at 90–100 dB produced significant BBB opening (23). There were no changes in the BBB integrity when the stimulus was 70 dB or when 1-h music exposure was used (23).

The repetitive mode of loud music adapts the brain to sound stress and protects its structure from sound-induced injuries. Indeed, we have found no appearance of apoptotic cells, morphological alterations in the brain tissues, or in the spiral ganglion cell 1 h after music impact (2-h intermittent exposure) when the BBB was opened and 4 h after music influences when the BBB integrity was fully restored, or 4 weeks after delayed music effects (23).

The frequencies range of music (Scorpions “Still Loving You”) is 11–10,000 Hz and maximal intensity is around 100 Hz (see SI in Ref 24). The hearing sensitivity in mice is between 1–90 kHz (28) and rats has the greatest sensitivity occurring between 8 and 38 kHz (29). Thus, the maximum intensity frequency of 100 Hz is infrasound (IS) for mice and rats. The IS-induced BBB opening has been shown in other experimental works (35, 36). However, there are no findings demonstrating direct IS effect on the BBB permeability in humans. There is a growing evidence that humans are indeed receptive to IS and that exposure to low-frequency sounds can give rise to high levels of distress, sleep disturbances, headache, and depression (37). However, Leventhall (38) claimed that “if you cannot hear a sound and you cannot perceive it in other ways and it does not affect you, p.135”. The World Health Organization suggests “there is no reliable evidence that SI below the hearing threshold produce physiological or psychological effects, p.4” (39).

### Spectrofluorometric assay of Evans Blue extravasation

Evans Blue dye (Sigma Chemical Co., St. Louis, Missouri, 2 mg/body weight, 1% solution in physiological 0.9% saline) was injected into the femoral vein and circulated in the blood for 30 min. Then, the rats were decapitated, their brains were quickly collected and placed on ice (no anticoagulation was used during blood collection). Prior to brain removal, the brain was perfused with saline to wash out the remaining dye in the cerebral vessels. The level of Evans Blue in the brain was evaluated in accordance with the recommended protocol (40) in the HG and HG+Music groups 21 days after their living in the individual cages under experimental condition as well as 21 days after implantation of glioma cell in the GBM and GBM+Music groups.

### Confocal microscopy of the BBB opening

Confocal microscopy was performed to confirm an increased BBB permeability to Evans Blue dye. Rats were decapitated and the brains were quickly removed, fixed with 4% neutral buffered formalin for 24 h, and cut into 50-µm thick slices on a vibratome (Leica VT 1000S Microsystem, Germany). The slices were incubated for one night with the goat anti-mouse NG2 antibody (1:500; ab 50009, Abcam, Cambridge, United Kingdom) and goat anti-rabbit GFAP antibody (1:500; ab 207165, Abcam, Cambridge, United Kingdom). In the next day, after several rinses in phosphate-buffered saline, the slices were incubated for 1h with 130 µl fluorescent-labeled secondary antibodies (goat anti-mouse IgG (H+L) Alexa Four 647; goat anti-rabbit IgG (H+L) Alexa Four 488; Invitrogen, Molecular Probes, Eugene, Oregon, USA). The slices were analyzed using a confocal microscope (Leica SP5, Germany). Approximately 8–12 slices per animal from cortical and subcortical (excepting hypothalamus and choroid plexus where the BBB is leaky) regions were imaged.

### Monitoring of distribution of fluorescent BZM and Omniscan in the rat brain

The Hamilton syringe (25 μl, Hamilton Co, Reno, Nevada, US) with the needle (25 Gauge) was mounted onto the injection pump using the stereotaxic manipulator (Stoelting, New York, US). A small cranial burr hole was drilled through the skull using a variable speed dental drill (with a 1-mm drill bit). The 5 μl of FITC-BZM or Omniscan^®^ at a rate of 1 μl/min was injected into the right lateral ventricle (AP-1.0 mm; ML−1.4 mm; DV-3.5 mm). The needle was left in the ventricle for 10 min and then removed at a rate of 1 mm/min to prevent the reflux of FITC-BZM or Omniscan^®^. The burr hole and scalp incision were closed with bone wax (Ethicon, Somerville, NJ) and with cyanoacrylate glue (Henkel Consumer Adhesive Inc. Scottsdale, Arizona), respectively.

The monitoring of the distribution of FITC-BZM or Omniscan^®^ was performed 3 h after the injection of tracers in two groups: (1) the healthy rats without music exposure (n=7) and (2) the healthy rats with the music-induced BBB opening (n=7). The optical imaging of FITC-BZM spreading was performed using homemade optical instrument based on monochrome camera acA2040-90um (Basler AG, Germany) and 50 mm 2,8 C-mount CCTV objective lens (Tamron, Japan). The lens was attached to the camera with 15-mm extension tube to ensure macro imaging with 23.3 to 31.8-mm field of view depending of the lens focusing ring adjustment. The lens was mounted on vertical manual translation stage (Standa, Lithuania) above a Petri dish where samples were submerged in buffer solution. The top surface of each sample was covered with 25×50×0.17 mm cover glass. The slider with filter sets (49019, 49002, Chroma Technology, USA) was placed just below objective lens. Each filter set was illuminated with homemade condensers with 1W LEDs (635 nm for 49019; and 460 nm for 49002) to ensure uniform illumination over the camera field of view. Led illuminators were synchronized with camera “fire” output.

The camera resolution was 2048×2048 pixels at 12-bit grayscale. Images were acquired in a dark room at constant exposure time of 200 ms and other settings kept unchanged for all samples. Image acquisition and processing were performed with custom software developed using N I Vision and LabVIEW software (National Instruments, USA) and FIJI open source image processing package (41). Image processing procedures were identical for each pair of images (control and music-treated sample) for each channel to ensure accurate comparison of fluorescence intensity.

The accumulation of MRI contrast agent Omniscan^®^ (0.1 mM/kg) in rat brain was studied using an ultra-high-field tomography BioSpec 117/16 USR (11 T, Bruker, US). Rats were anaesthetized with 2% isoflurane at 1 L/min N_2_O/O_2_—70:30 (Isoflurane, Baxter Healthcare Corp., USA) using an anesthesia machine (The Univentor 400 Anesthesia Unit, Univentor, Malta). The temperature of animals was maintained using a water circuit in a tomographic bed-table with a surface temperature of 30°C. A pneumatic breathing sensor (SA Instruments, Stony Brook, N.Y., US) was placed under the lower torso, which made it possible to control the depth of anesthesia.

Information on the distribution of contrast in the rat brain was investigated using T1-weighted images obtained using the RARE method (Rapid Acquisition with Relaxation Enhancement). Pulse sequence parameters of the method (TE = 10 ms, TR = 400 ms), image parameters (size 1.8 × 1.8 cm; matrix 256 × 256 points; slice thickness 0.5 mm; voxel dimensions 75 µm x 75 µm x 0.5 mm; the distance between the slices is 0.5 mm; the number of slices is nine; the orientation of the slices is coronal), the total scanning time was 7 min.

The accumulation of MRI contrast was expressed as the ratio of the level of the MRI signal in the studied structures to the level of the MRI signal in the reference, which was a phosphate-buffered microtube (0.5 ml) placed along the head of the mouse. The MRI scans were processed using the ImageJ software.

### Quantitative analysis of confocal images

The analysis of fluorescence of BZM in the brain slices was carried out on a fluorescence microscopic system described above. For the quantitative analysis of intensity signal from BZM, ImageJ was used for image data processing and analysis. The intensity of fluorescence for each slide was integrated over rectangular ROIs bounding brain slice. The integral value was divided by slice area. The areas of brain slices were calculated using the plugin “Analyze Particles” in the “Analyze” tab, which calculates the total area of fluorescence intensity tissue elements—the indicator “Total Area”. In all cases, 10 regions of interest were analyzed.

### Immunohistochemistry and confocal imaging

For the Immunohistochemistry (IHC) analysis, the brain tissues were collected and free-floating sections were prepared. The brains were fixed for 48 h in a 4% saline solution-buffered formalin, then sections of the brain with a thickness of 40–50 microns were cut on a vibrotome (Leica, Germany). Sections were processed according to the standard IHC protocol. The sections of mouse brain were imaged using a Leica SP8 confocal laser scanning microscope (Leica, Germany).

For IHC assay rats were euthanized with an intraperitoneal injection of a lethal dose of ketamine and xylazine and intracardially perfused with 0.1 M of PBS for 5 min. Afterward, the brains were removed and fixed in 10% buffered formalin, wiring material in alcohols, pouring into paraffin. Paraffin sections were stained with hematoxylin and eosin and IHC studies were performed using the REVEAL Polyvalent HRP-DAB Detection System. Monoclonal antibody Abcam (USA): bax (clone 100/D5, ab692) and Ki67 (clone SP6, ab16667) were used at a dilution of 1:100 to the antibody. When staining with IHC markers, positive and negative controls were used to exclude false-negative and false-positive results to create standardization of staining conditions and increase the objectivity of the results. The percentage of positively expressing cells in 10 fields of view of each sample and the intensity of immunohistochemical reactions (weak, moderate, and pronounced) was calculated. All studies were performed using a MicroVisor of medical transmitted light µVizo-103 (LOMO, Russia) with a magnification of 774. The quantitative analysis of immunopositive cells (%) expressed of Bax (apoptosis) and Ki67 (proliferation) was performed in the HG, GBM, GBM+Music, GBM+BZM, GBM+BZM+Music groups, n=7 in each group.

### Statistical analysis

The results are presented as the mean ± standard error of the mean (SEM). The data partially did not follow the normal distribution and were not heteroscedastic. Differences from the initial level in the same group were evaluated by the Wilcoxon test. Intergroup differences were evaluated using the Wilcoxon rank sum test with continuity correction (Mann–Whitney–Wilcoxon test). The significance level was set at p < 0.05 for all analyses, n=7 in each group.

## Results

### The music-induced BBB opening increases BZM distribution to the brain

The important focus on the GBM progression through recruitment of the normal cerebral vessels with an intact BBB is one of most dominant in GBM research and clinical practice (7). The intact BBB is not permeable for the high-molecular-weight antibodies, such as BZM (149 kDa), which might be a major reason of low therapeutic effectiveness of BZM. Therefore, we hypothesized that the music-induced BBB opening can significantly improve BZM distribution to the brain.

In the first step, we performed the quantitative analysis of the BBB opening to Evans Blue dye (961 Da) that is a commonly used tracer for the evaluation of the BBB disruption (23, 24, 42–44). This tracer is non-toxic and not metabolically active dye. After intravenous injection, Evan Blue dye binds to serum albumins creating the high-molecular-weight Evans Blue-albumin complex (EBAC, 68.5 kDa), which cannot cross an intact BBB.

**Figure 2:**
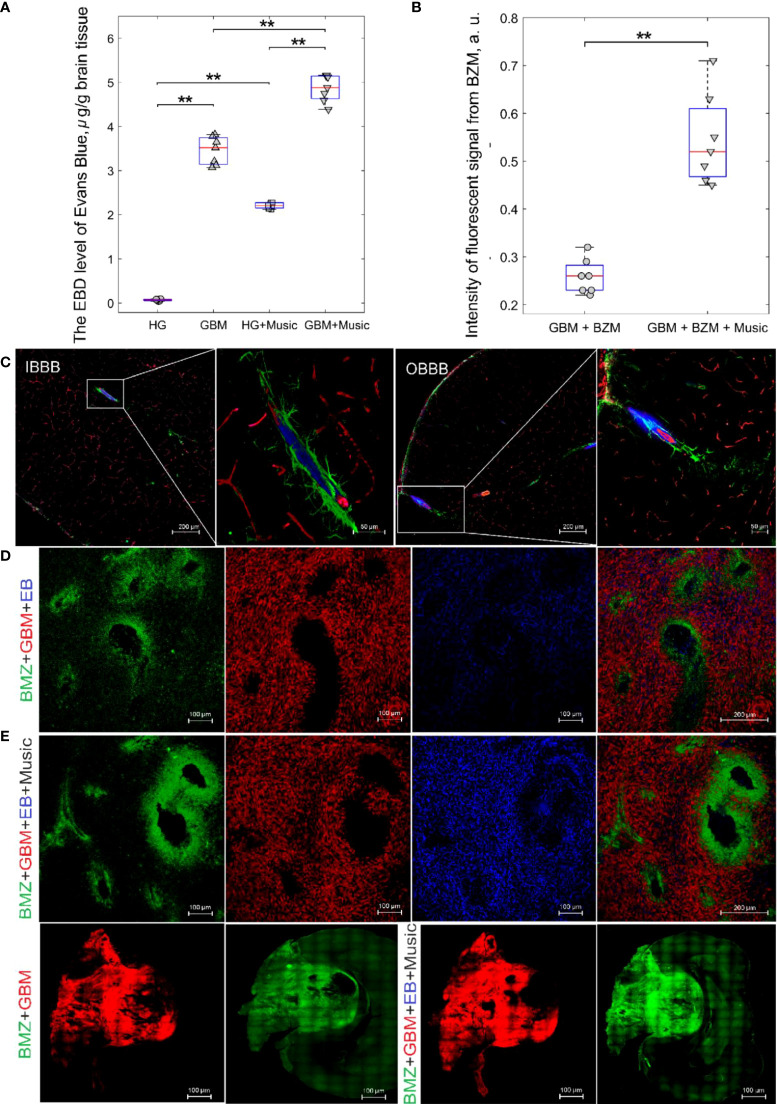
The music-induced BBB opening for EBAC and FITC-BZM in rats: **(A)** the EBAC quantitation before and after music impact in the HG, HG+Music, GBM, GBM+Music groups, ** - p<0.01, the Mann–Whitney–Wilcoxon test, n=7 in each group; **(B)** Quantification of the intensity of the fluorescent signal from BZM in the GBM+BZM group and the GBM+BZM+Music group, ** - p<0.01, the Mann–Whitney–Wilcoxon test, n=7 in each group; **(C)** Confocal imaging of music-induced BBB opening for EBAC (blue) using the markers of pericytes (NG2, red) and astocytes (GFAP, green) in rats from HG group, the intact BBB (IBBB) and the opened BBB (OBBB); **(D)** Representative confocal images of distribution of BZM (green) in GBM (red) tissues of rats from the GBM+BZM and the GBM+BZM+Music groups; **(E)** - a whole section of brain imaging from the BZM+GBM and the BZM+GBM+Music groups.

**Figure 2A** demonstrates the EBAC quantitation. The BBB permeability to EBAC was 31.3-fold higher (3.45 ± 0.21 µg/g tissue *vs*. 0.11 ± 0.06 µg/g tissue, p<0.001, the Mann–Whitney–Wilcoxon test, n=7 in each group) in rats with GBM (21 days after implantation of glioma cells) *vs*. the healthy animals suggesting the BBB disruption in the brain tumor.

Music caused the BBB opening for EBAC in healthy rats (2.21 ± 0.12 µg/g tissue *vs*. 0.11 ± 0.06 µg/g tissue, p<0.001, the Mann–Whitney–Wilcoxon test, n=7 in each group) and increased by 1.4 times the EBAC leakage in rats with GBM (4.86 ± 1.30 µg/g tissue 3.45 ± 0.21 µg/g tissue, p<0.001, the Mann–Whitney–Wilcoxon test, n=7 in each group). **Figure 2C** demonstrates confocal imaging of music-induced BBB opening for EBAC in cortical and subcortical regions of the brain using the markers of pericytes and astrocytes in rats from HG group. In our previous work, we demonstrated music-induced BBB opening in 11 brain regions, including frontal, parietal, occipital, temporal, cingulate cortex, hypothalamus, thalamus, hippocampus, inferior and superior colliculi, cerebellum, pons, and medulla (23).

In the next step, we analyzed the delivery of FITC-BZM into the brain of rats with GBM without and after the music-induced BBB opening. **Figures 2B**, **D** illustrate that distribution of BZM around the cerebral vessels was 2.1-fold higher in the GBM+BZM+Music *vs*. the GBM+BZM groups (0.54 ± 0.04 a.u. *vs*. 0.25 ± 0.05 a.u., p<0.001, the Mann–Whitney–Wilcoxon test, n=7 in each group). **Figure 2E** shows a whole section of brain imaging from the BZM+GBM and the BZM+GBM+Music groups. The intensity of signal from BZM in the GBM mass and in adjacent regions of brain tissues was higher in the BZM+GBM+Music group vs. the BZM+GBM group.

Thus, this series of experiments clearly demonstrates that music-induced BBB opening increases the delivery of EBAC into the brain tissues and the extravasation of BZM into the brain around the cerebral vessels of rats with GBM.

### Music-induced BBB opening increases drainage of the brain tissues and BZM distribution to the brain fluids

The interstitial fluid (ISF) flow is an important route of drug delivery into the brain (45). The brain tumors are associated with high interstitial pressure that is main reason of the failure of many potentially effective therapeutic approaches (46). The disorders of drainage of ISF seen in GBM (47) can explain the inability to effectively deliver and sustain adequate drug concentrations within the brain (48). Therefore, the development of methods for modulation of ISF-based drug delivery have considerable interest (45).

**Figure 3:**
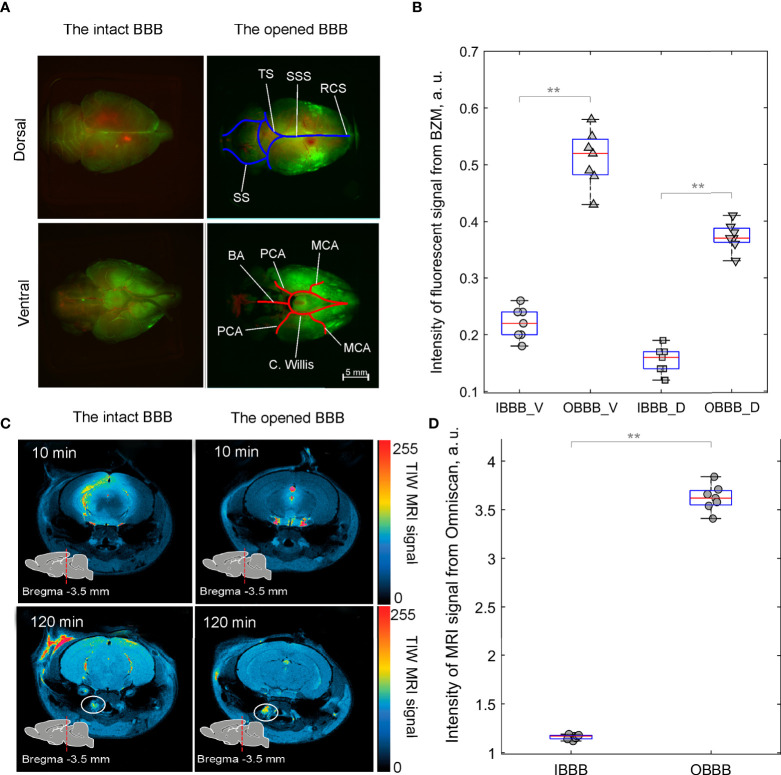
Distribution of FITC-BZM (green) and Omniscan (red, yellow and blue colors indicate intense, weak and poor MRI signal from Omniscan, respectively) in healthy rats before and after music-induced BBB opening: **(A)** Images of perivascular distribution of FITC-BZM on the dorsal and ventral surfaces of the brain with the intact BBB and the opened BBB. Rats were euthanized 3 h after the intraventricular injection of FITC-BZM. The BBB was opened after the intraventricular injection of FITC-BZM (2 h of listening to music and 1 h of delayed time of BBB opening). Images are representative of n=7 rats. MCA, Middle cerebral artery; **(C)** Willis, Circle of Willis; PCA, Posterior cerebral artery; ACA, Anterior cerebral artery; BA, Basilar artery; SSS, Superior sagittal sinus,; SS, Sigmoid sinus; TS, Transverse sinus; RCS, Rostral confluence sinus. **(B)** Quantification of the intensity of the fluorescent signal from FITC-BZM in rats with the intact (IBBB) and the opened BBB (OBBB), V—ventral aspect of the brain and D—dorsal aspect of the brain, **p<0.01, the Mann–Whitney–Wilcoxon test, n=7 in each group. **(C)** Representative MRI images of distribution of Omniscan in the brain and clearance of tracer into dcLNs (circled). **(D)** Quantification of the intensity of MRI signal from Omniscan in rats with IBBB and OBBB, **p<0.01, the Mann–Whitney–Wilcoxon test, n=7 in each group.

In our previous results, we demonstrated that sound-induced BBB opening is associated with an activation of lymphatic drainage of the brain tissues (23, 24). Based on these data, we studied effects of music-induced BBB opening on the distribution of FITC-BZM in the brain fluids after its injection into the right lateral ventricle as a communicating cavity of fluid drainage in healthy rats using *ex vivo* fluorescence microscopy. **Figures 3A, B** illustrate that the BZM fluorescence was higher in rats with the opened BBB *vs*. the intact BBB with stronger distribution of FITC-BZM on the ventral than on the dorsal aspect of the brain (0.51 ± 0.04 a.u. *vs*. 0.22 ± 0.03 a.u., p<0.001, the Mann–Whitney–Wilcoxon test in ventral aspect of the brain and 0.37 ± 0.07 a.u. *vs*. 0.15 ± 0.02 a.u., p<0.001, the Mann–Whitney–Wilcoxon test in dorsal aspect of the brain, n=7 in each group). These data suggest that the BBB opening contributes the distribution of FITC-BZM from the ventricle to the ventral area of the brain, where are localized of the basal meningeal lymphatic vessels playing an important role in the brain drainage (49). Additionally, we studied in *in vivo* experiment the effects of music on the brain drainage processes using real-time MRI imaging of distribution of contrast agent (Omniscan) in the brain tissues of healthy rats. Our data demonstrated that the BBB opening significantly increased the distribution of Omniscan from the right lateral ventricle into the deep cervical lymph nodes (dcLNs) (3.62 ± 0.08 a.u. *vs*. 1.16 ± 0.06 a.u., p<0.001, the Mann–Whitney–Wilcoxon test, n=7 in each group) (**Figures 3C, D**).

These findings suggest that music significantly increases distribution of tracers (FITC-BZM and Omniscan) in the rat brain from the right lateral ventricle through the pathways of brain drainage system (perivascular and lymphatic), which are an important route of drug delivery into the brain (45).

### Music-induced BBB opening improves therapeutic effects of BZM in rats with GBM

**Figure 4:**
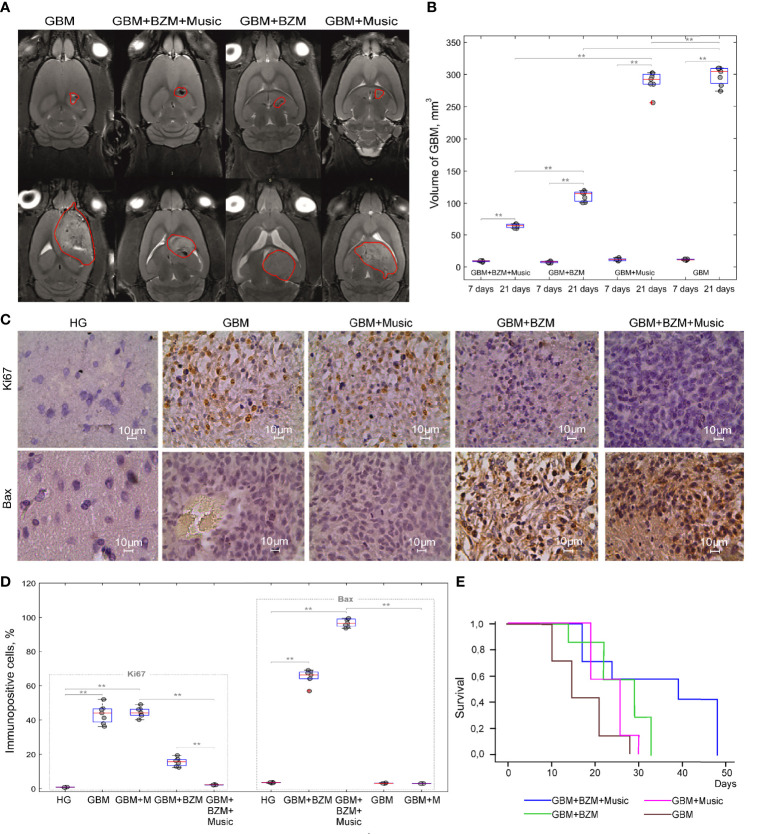
The effects of BZM on the GBM growth and survival in rats with the intact and the opened BBB: **(A)** Representative MRI images of GBM in the GBM, GBM+Music, GBM+BZM, and GBM+BZM+Music groups; **(B)** The MRI analysis of the GBM volume in the GBM, GBM+Music, GBM+BZM, and GBM+BZM+Music groups, the Mann–Whitney–Wilcoxon test, **p<0.01, n=7 in each group; **(C)** The ICH assay of expression of Ki67 (marker of proliferation) and Bax (marker of apoptosis) in the HG, GBM, GBM+BZM, GBM+Music, and GBM+BZM+Music groups; **(D)** The quantitative analysis of immunopositive cells (%) expressed Bax and Ki67 in the HG, GBM, GBM+BZM, GBM+Music, and GBM+BZM+Music groups, the Mann–Whitney–Wilcoxon test, **p<0.01, n=7 in each group; **(E)** Kaplan–Meier overall survival plots in the GBM, GBM+BZM, GBM+Music, and GBM+BZM+Music groups, n=7 in each group. GBM, glioblastoma; BZM, bevacizumab; HG, healthy group.

In the final step, we analyzed the therapeutic effects of BZM on the glioma growth without and with the music-induced BBB opening. **Figures 4A, B** demonstrate that the GBM volume was significantly increased from 7 to 21 days of observation in all tested groups. The BZM suppressed the glioma growth that was more pronounced in rats listening to music compared to rats not listening to music. Indeed, the GBM volume on 21 days of tumor progression was increased 7.1-fold (64.1 ± 3.3 mm^3^ *vs*. 9.0 ± 1.1 mm^3^, p<0.001, the Mann–Whitney–Wilcoxon test, n=7 in each group) in the GBM+BZM+Music group and 14.4-fold (111.3 ± 9.2 mm^3^ *vs*. 7.7 ± 2.2 mm^3^, p<0.001, the Mann–Whitney–Wilcoxon test, n=7 in each group) in the GBM+BZM group. Thus, the GBM volume on 21 day of tumor growth was 1.7-fold less in the GBM+BZM+Music *vs*. the GBM+BZM groups (64.1 ± 3.3 mm^3^ *vs*. 111.3 ± 9.2 mm^3^, p<0.001, the Mann–Whitney–Wilcoxon test, n=7 in each group).

The additional comparison of the tumor volume on 21 days of glioma growth between the groups receiving and not receiving BZM revealed that the GBM volume was 4.5-fold less in the GBM+BZM+Music *vs*. the GBM+Music groups (64.1 ± 3.3 mm^3^ *vs*. 288.5 ± 16.2 mm^3^, p<0.001, the Mann–Whitney–Wilcoxon test, n=7 in each group) and was less 2.6-fold in the GBM+BZM *vs*. GBM groups (111.3 ± 9.2 mm^3^ *vs*. 297.6 ± 12.8 mm^3^, p<0.001, the Mann–Whitney–Wilcoxon test, n=7 in each group).

There were no differences in the GBM volume on 21 days of glioma growth between the GBM and GBM+Music groups. So, the GBM volume was increased 25.8-fold in the GBM group (297.6 ± 12.8 mm^3^ *vs*. 11.5 ± 2.3 mm^3^, p<0.001, the Mann–Whitney–Wilcoxon test, n=7 in each group) and 24.8-fold in the GBM+Music group (288.5 ± 16.2 mm^3^ *vs*. 11.6 ± 1.8 mm^3^, p<0.001, the Mann–Whitney–Wilcoxon test, n=7 in each group).

These findings demonstrate significantly less glioma progression in rats receiving BZM with music compared with other groups (**Figure 4B**).

**Figures 4C, D** illustrate the cellular mechanisms of BZM effects on the glioma cells. The glioma growth was characterized by an increased expression of Ki67 (a marker of cell proliferation) *vs*. the normal brain suggesting the high proliferation of tumor cells that was similar between the GBM and GBM+Music groups (43.22 ± 8.40% *vs*. 0.81 ± 0.09%, p<0.001 for the GBM group and 41.44 ± 6.51% *vs*. 0.81 ± 0.09%, p<0.001 for the GBM+Music group, the Mann–Whitney–Wilcoxon test, n=7 in each group). However, the Ki67 expression was significantly decreased in the groups receiving BZM that was more pronounced in rats listening additionally music compared with animals without music impact. Indeed, the expression of Ki67 was 18.7-fold lower in the GBM+BZM+Music *vs*. the GBM+Music groups (2.21 ± 0.22% *vs*. 41.44 ± 6.51%, p<0.001, the Mann–Whitney–Wilcoxon test, n=7 in each group) and 2.8-fold lower in the GBM+BZM *vs*. the GBM groups (15.40 ± 4.62% *vs*. 43.22 ± 8.40%, p<0.001, the Mann–Whitney–Wilcoxon test, n=7 in each group). The comparison of music effects on the Ki67 expression in rats with glioma receiving and not receiving BZM shows that the glioma proliferation was 6.9-fold less in the GBM+BZM+Music *vs*. the GBM+BZM groups (2.21 ± 0.22% *vs*. 15.40 ± 4.62%, p<0.05, the Mann–Whitney–Wilcoxon test, n=7 in each group).

The BZM induced apoptosis in the tumor cells with the high expression of Bax (a marker of apoptosis) in both groups of rats with and without music impact (96.32 ± 7.33% *vs*. 1.57 ± 0.07%, p<0.001 for the GBM+BZM+Music and 64.12 ± 3.31% *vs*. 1.57 ± 0.07%, p<0.001 for the GBM+BZM, the Mann–Whitney–Wilcoxon test, n=7 in each group). The comparison between these two groups showed that the BZM-mediated apoptosis in the glioma cells was 1.5-fold higher in the GBM+BZM+Music group *vs*. the GBM+BZM group (96.32 ± 7.33% *vs*. 64.12 ± 3.31%, p<0.001, the Mann–Whitney–Wilcoxon test, n=7 in each group). We did not observe an increase in the Bax expression in the groups not receiving BZM or in the HG.

These data suggest that the music-induced BBB opening improves the suppressive effects of BZM on the glioma volume and the cellular mechanisms of tumor progression that was accompanied by higher survival among rats in the GBM+BZM+Music group *vs*. other groups [p=0.008, X2 test Log Rank (Mantel-Cox) = 9,171, Kaplan-Meier method] (**Figure 4E**).

## Discussion

The development of the new methods for the effective distribution of drugs across an intact BBB is a crucial step in the effective therapies for GBM and is most required knowledge in further consideration of any clinical trials for a newly diagnosed or recurrent GBM (2). The existing concept of GBM as a whole-brain disease where the fast GBM progression is directly linked to the prevalent recruitment of the normal cerebral vessels by the glioma tissue (7). Due to high weight molecular antibody (149 kDa), BZM has comparably low penetration rate for an intact BBB, which might be a major reason why its anti-tumor therapeutic ability necessitates further enhancement. Therefore, in this pilot work on rats, we studied the music-induced BZM distribution to rat brain with GBM and analyzed the progression of brain tumor in the treated and untreated groups with anti-VEGF antibody and the music-induced BBB opening.

Our results revealed that music-induced BBB opening increased substantially BZM distribution to the brain around the cerebral vessels of rats with GBM. We assume that loud music temporally modulates the permeability of both intact and tumor BBB that is associated with the effective BZM distribution in ISF of the brain and along the normal cerebral vessels.

To test this hypothesis, we studied the distribution of FITC-BZM in ISF as an important route of drug delivery into the brain (45). We, therefore, studied spreading of FITC-BZM from the right lateral ventricle to the brain surface in healthy rats with and without music exposure. The results revealed that music-induced BBB opening increased distribution of FITC-BZM more significantly on the ventral than on the dorsal aspects of the normal brain. So, after intraventricular injection, FITC-BZM distributed more intensively along the cerebral arteries within the subarachnoid space in the region of the circle of Willis at the base of the brain with spreading within this space along the middle cerebral artery and its branches than along the main venous sinuses, such as the superior sagittal, sigmoid, and transverse sinuses. Using real-time MRI analysis of Omniscan distribution from the right lateral ventricle, we observed clearance of tracer from the brain into dcLNs that was much faster in rats with the opened BBB *vs*. rats with an intact BBB. These data suggest that music-induced BBB opening contributes effective distribution of tracers (BZM and Omniscan) through the brain fluids and lymphatic system. The obtained results are consistent with our previous data suggesting that sound-induced BBB opening is associated with activation of lymphatic drainage of the brain tissues (23, 24). The ISF flow is an important route of drug delivery into the brain (45). The music-mediated distribution of BZM through brain fluids might be a crucial approach leading to enhancement of therapeutic effect of anti-VEGF antibody.

In fact, we found that the proliferation of glioma cells was completely suppressed in the GBM+BZM+Music group and was only decreased in the GBM+BZM group. Meanwhile, BZM was able to promote the apoptosis of tumor cells, which were greater in rats with GBM and received BZM with music *vs*. rats with GBM and received BZM alone. The MRI analysis of GBM volume clearly demonstrated much slower tumor growth in the GBM+BZM+Music group than in the GBM+BZM group. Therefore, the BZM treatment together with the music-induced BBB opening led to more effectively suppression of GBM progression than application of BZM along.

Since loud music is a stress factor in our previous study (23), we have present the mechanisms underlying music-induced BBB opening. The stress hormone epinephrine induces the BBB opening *via* relaxation of the cerebral microvessels that is accompanied by an increase in gaps between the tight junction (TJ) proteins and the changes in ultrastructure of the astrocytes (50–53). The sound-induced increase in the level of stress hormones were demonstrated in humans and animals (54). The loud music causes a rise of serum epinephrine with a significant increase in the cerebral blood flow (CBF) in both macro- and micro-circulatory levels (23). The elevation of epinephrine by stress induces an increase of CBF associated with the changes in the tone of cerebral vessels that might be an initial factor triggering the BBB opening. Indeed, previously, we reported that 1 h after music exposure was accompanied by disorganization of the TJ assembly (23). However, the sound-induced BBB opening is characterized by fast recovery of the BBB integrity. We suppose that during music/sound BBB opening, the TJ proteins are internalized that can be a reason of a temporal loss of their surface on the endothelial cells (23). However, it is impossible to estimate the precise time of BBB recovery because we observed the high variability of the BBB closing in different rodents. The BBB recovery can start already during stress *via* a decrease in the BBB permeability induced by corticosterone (55).

In our previous study (23), we did not find the acute and delayed brain injuries and the cochlea after the music-induced BBB opening. However, even short-lasting and reversible increase in the BBB permeability can be harmful for the brain due to the BBB opening to viruses, bacteria, and toxins. Nevertheless, there is a large potential risk for the group of the BBB opening, including professional musicians, teenagers, and people, which listen loud music in concerts, nightclubs and headphones. Therefore, further studies of the music-induced BBB opening are required for investigations in humans. Notice, that focused ultrasound (FUS) is the most promising tool for the BBB opening in humans (56, 57). However, the FUS-induced BBB opening is accompanied by the development of sterile inflammation (58) and not effective without micro-bubbles (59) that is additional limitation of this method for people with allergy, vascular pathologies, and high risk for thrombosis that is often problem for oncological patients (60). Thus, the music-induced BBB opening for patients, who can listen loud music, might be a good alternative approach for brain drug delivery.

The music opens BBB in a non-specific manner that can be very important for therapy of GBM. Indeed, GBM grows through the normal cerebral vessels with an intact BBB that limits a drug delivery into the brain regions with newly formed tumor. Therefore, the effective pharmacological therapy of GBM will only be possible if the invasive brain tumor branches will be adequately treated (3, 4). In this aspect, the non-specific opening of BBB by music can give more chances to deliver anti-tumor drugs into new branches of GBM that are overlooked in clinically used diagnostic methods.

The loud music has a high potential for clinical applications as new method for the therapy of Alzheimer’s disease (AD), which is the whole-brain disease characterized by diffusive injuries of different brain areas. The FUS-induced opening of BBB without pharmacological treatment causes a decrease in beta-amyloid plaque in the brain of patients with AD that is accompanied by improvement of their neurological status (56). It is assumed that this may be due to the activation of the lymphatic drainage function of the brain after the BBB opening that we observed in our previous (23, 24) and current research. The search for methods to stimulate the drainage processes of the brain, aimed at removing beta-amyloid from the brain fluid, is considered as a promising direction in the development of effective therapy for AD (61). The music/sound-induced BBB opening can be an alternative method to FUS in activation of beta-amyloid removing from the brain of patients with AD. The Parkinson’s disease (PD) is also accompanied by impaired meningeal lymphatic drainage in patients (62). The development of new technologies, probably using music, for the stimulation of removing of α-synuclein from the brain is considered as a perspective future therapy of PD.

Thus, music causes short-lasting and reversible increase in gaps between TJ without their injuries. There is an evidence that it might be due to stress-induced temporal internalization of TJ associated with the modulation of tone of micro-vessels by hormones, such as epinephrine and corticosterone. The BBB opening is accompanied by activation of lymphatic drainage of the brain tissues and an increase in the ISF movement, which is an important platform for drug delivery into the brain (45, 46). The phenomenon of music-induced BBB opening is observed in healthy subjects and in the normal cerebral vessels, i.e., this process is physiological without any injuries of the brain structures and functions (23). We hypothesized that music improves the therapeutic effects of BZM *via* the BBB opening in the normal cerebral vessels and lymphatic drainage of the brain tissues. This contributes better distribution of BZM in the brain fluids and among the normal cerebral vessels, which are used by GBM for invasion and co-opt existing vessels as a satellite tumor form.

In this study, we did not analyze the CSF barrier permeability because we were aimed for analysis of the effects of music on the BBB opening and the BZM delivery into the brain tissues. However, we found that music significantly increases distribution of tracers (FITC-BZM and Omniscan) in the rat brain from the right lateral ventricle through the pathways of brain drainage system. We hypothesize that music-mediated modulation of cerebrovascular barriers is not specific and music-induced increase in the permeability of the choroid plexus epithelial cells, which are leakier than the BBB, can be another important mechanisms underlying music-based drug delivery into the brain.

Obviously, not only the music of Scorpions “Still loving you” is effective for opening of the BBB. The usage of only one rock ballad limits our study from the point of view of studying the effects of different types of music on the BBB, but gives advantages in data reproducibility. Since the aim of our research was to study the possibility of improving the delivery of BZM to the brain tissue using music, we used an established and published protocol (23, 63). For mice, loud music is highly stressful. Therefore, the reaction to loud music, as to stress, will be the same. On the contrary, for humans, the emotional effects of music is of great importance. Therefore, despite the fact that potentially different rock ballads with an intensity of 100 dB can open the BBB, we do not exclude the dependence of effectiveness of music-induced BBB opening on the emotions that music evokes. The further studies of the various music effects (rock, jazz, classical, etc.) on the BBB permeability in humans will shed light on the applicability of this method in clinical practice, its reproducibility in different individuals, and safety.

The BZM is usually used together with TMZ in the clinic. We only start the first step with BZM using its fluorescent form and optical methods for the analysis of the Bev distribution in the brain tissues. The investigation of music effects on BZM+TZM, as combination of anti-VEGF and cell targeted therapy, is a next logical step for our understanding of the clinical relevance of music for treatment of brain tumor. The TMZ has small size and lipophilic properties. Therefore, TMZ is able to cross the BBB. However, this concentration in the brain is approximately 30% of plasma concentration (64). Since, the tumor BBB is leaky, most of TMZ concentrations enter GBM mass. The music-induced BBB opening can contribute for better distribution of TMZ in the healthy brain areas, where new branches of GBM migrates. Furthermore, the development of resistance to TMZ often becomes the limiting factor in effective treatment of GBM (65). The recent study suggests that BZM may affect chemotherapeutic delivery through alteration of perfusion dynamics, suggesting that BZM may play a role in TMZ delivery and preventing resistance (67). Thus, the music-mediated improvement of BZM therapeutic effects can contribute to prevent TMZ resistance.

By cutting off the biological activity of VEGF, BZM will bring about ischemia and hypoxia of the tumor cells and thus affect the growth, the attack and the proliferation of a cancer (67–69). According to the latest research (70), VEGF can adjust cells’ endoplasmic reticulum stress (ERS) by way of directly activating this signal channel. Thus we can speculate that the reason why BZM will play a role in treating GBM is that it can accelerate cell apoptosis through the mechanism of ERS. Our finding suggestion are consistent with other results reflecting apoptotic activity VEGFs, including BZM (71–73). The anti-proliferation and apoptotic effects of BZM on the GBM cells were enhanced by music. Thus, the BBB opening alone by music might contribute to raise sensitivity of BZM to the GBM cells *via* improving BZM distribution in the brain tissues, and especially along the cerebral vessels with the intact BBB, which used by GBM for its spreading.

## Conclusions

In sum, the music-induced BBB opening increases the suppressive effects of BZM on the GBM volume and the cellular mechanisms of tumor progression that was accompanied by higher survival among rats in the GBM+BZM+Music group *vs*. other groups. These findings open the new perspectives for an improvement of therapeutic effects of BZM *via* the music-induced BBB opening for BZM in the normal cerebral vessels, which are used by GBM for invasion and co-opt existing vessels as a satellite tumor form. Despite the fact that music opens BBB in a non-targeted manner, music has a high potential for clinical applications as an easily used, non-invasive, low cost, labeling free, perspective, and completely new approach for the treatment of GBM, which has diffusive growth. We address to further research of the music-induced BBB opening as a proof of concept for exploration of sound-induced drug delivery to the brain. Such issues as the study of efficacy of the drug delivery into the brain and the effect of different levels of sound on the pharmacokinetics of the delivered drugs can shed light on the development of a new alternative method of drug brain delivery for brain oncology, different neurodegenerative diseases, and brain trauma.

## Data availability statement

The raw data supporting the conclusions of this article will be made available by the authors, without undue reservation.

## Ethics statement

All experimental procedures were performed in accordance with the “Guide for the Care and Use of Laboratory Animals”, Directive 2010/63/EU on the Protection of Animals Used for Scientific Purposes, and the guidelines from the Ministry of Science and High Education of the Russian Federation (№ 742 from 13.11.1984), which have been approved by the Bioethics Commission of the Saratov State University (Protocol No. 7).

## Author contributions

OS-G and JK initiated and supervised this work. AS, AT and AD performed confocal analysis. IF performed analysis of distribution of FITC-BZM in the brain. AK made MRI scans. SD, DoE, EA and KA made synthesis of BZM. NN performed IHC analysis. IB, AE, DaE and VA performed most of the experiments. OS-G and JK reviewed all results and wrote the manuscript. JK participated in the discussions of the results and commented on the manuscript. All authors contributed to the article and approved the submitted version.

## Funding

OS-G, AK, IF, AD, IB, AT, AE, GK, VT and JK were supported by RF Governmental Grant No. 075-15-2022-1094, Grant from RSF No. 20-15-00090; 21-75-10088, Grant from RFBR 20-015-00308-a; China-a 19-515-55016.

## Acknowledgments

We thank research center "Symbiosis" and immunochemistry laboratory IBPPM RAS for their support with immunofluorescence analysis and confocal microscopy within Project No. GR 121031100266-3.

## Conflict of interest

The authors declare that the research was conducted in the absence of any commercial or financial relationships that could be construed as a potential conflict of interest.

## Publisher’s note

All claims expressed in this article are solely those of the authors and do not necessarily represent those of their affiliated organizations, or those of the publisher, the editors and the reviewers. Any product that may be evaluated in this article, or claim that may be made by its manufacturer, is not guaranteed or endorsed by the publisher.
